# A New Detection Method for the Biomarkers of Soybean Isoflavones in Human Urine on the Basis of Packed‐Fiber Solid‐Phase Extraction

**DOI:** 10.1002/fsn3.71116

**Published:** 2025-10-30

**Authors:** Lanling Chu, Yuqi Dai, Qinghai Hu, Erzheng Su, Shuo Qi, Xiaoman Jiang, Anni Fu, Qianqian Jiang, Xuejun Kang

**Affiliations:** ^1^ State Key Laboratory for Development and Utilization of Forest Food Resources Nanjing Forestry University Nanjing PR China; ^2^ Yantai Key Laboratory of Special Medical Food (Preparatory), School of Food and Biological Engineering Yantai Institute of Technology Yantai PR China; ^3^ School of Biological Science and Medical Engineering Southeast University Nanjing PR China

**Keywords:** biomarkers, detection, electrospinning nanofibers, isoflavones, solid‐phase extraction

## Abstract

Traditional dietary assessment methods such as 24‐h dietary recall methods are not accurate enough, and the biomarkers to objectively evaluate food consumption have become attractive recently. Soy isoflavones are a typical plant‐based estrogen that helps to slow menopause symptoms, helping to lower cholesterol levels and reduce the risk of arteriosclerosis, and studies have shown a significant correlation between urinary isoflavones excretion and soy intake, which can be used as a useful biomarker. Because of the complex urine sample matrix and the large range of isoflavone concentration, it is vital to develop a new detection method to decrease the impurities interference and increase the target concentration for accurate detection of urine isoflavones. In this study, a few types of nanofibers were prepared and utilized as solid‐phase extraction adsorbents for sample pretreatment, and the homemade packed‐nanofiber solid‐phase extraction columns were chosen to purify the matrix and concentrate the analytes. A new method for rapid detection of soybean isoflavones in urine on the basis of packed‐fiber solid‐phase extraction combined with ultraviolet spectrophotometer was established, which provides an efficient reference for assessment of individual dietary intake.

## Graphical abstract

Electrospun nanofibers were prepared and utilized as solid‐phase extraction adsorbents for sample pretreatment. Homemade packed‐nanofiber solid‐phase extraction columns were created to purify the urine matrix and concentrate the analytes; that is, packed‐fiber solid‐phase extraction (PFSPE). A new method for rapid detection of the biomarkers of soybean isoflavones in urine on the basis of PFSPE combined with ultraviolet spectrophotometry was established, which provides an efficient reference for assessment of individual dietary intake.

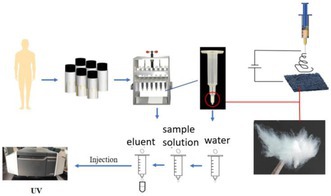

## 1 Introduction

Dietary intake of beans has been recognized with the reduced risk of chronic cardiovascular disease, as well as improvements in coronary heart disease (Naghshi et al. 2024), and the benefits of regular soy intake have been epidemiologically proven (Duan et al. 2022). Research on the relationship between the intake of specific foods and human health requires very accurate information on dietary intake, and how to obtain accurate and reliable data on dietary intake is a key challenge (Willis et al. 2020). However, traditional data collection on food frequency questionnaire and 24‐h dietary recall method requires training of investigators, and the survey results are more dependent on short‐term memory, which does not signify accurate intake (Sun et al. 2022). Therefore, it is urgent to find a more objective intake measurement method. There are long‐term exposure studies showing that the level of biomarkers in blood or urine can objectively assess food consumption, which will be a complementary tool to replace traditional dietary assessment methods (Gormley et al. 2020). It is critical to advance the field of dietary biomarkers and produce reliable, repeatable levels of biomarkers that can be used in public health and clinical research.

Soybean isoflavones are a class of flavonoid compounds mainly found in soybeans and soy products, whose chemical structure is very similar to that of human endocrine estrogen estradiol, which can show biological activity similar to estrogen under certain conditions, and has a good regulation and improvement effect on endocrine and metabolic system diseases (Lei et al. 2024). They can prevent cardiovascular diseases, delay aging, reduce the incidence rate of breast cancer and prostate cancer, relieve menopause syndrome and osteoporosis caused by decreased estrogen secretion, and provide antioxidant and cholesterol reduction effects (Geng et al. 2022; W. Li, Twaddle, et al. 2023; X. B. Wang, Zhang, et al. 2023). In addition, soy isoflavones can also play an immune role by regulating the secretion of various cytokines (Goja et al. 2023). Importantly, soy intake is positively correlated with the levels of isoflavones in the urine, plasma, or serum of different populations (Jang et al. 2021), and in a traditional Japanese diet study, the excretion of soy isoflavones was positively correlated with the intake of soy and soy products (Adlercreutz et al. 1991). The total amount of daidzein, genistein, and glycosides in urine was also associated with the daily intake of soy‐based foods. Studies have also confirmed that the level of soy isoflavone biomarkers in morning urine is associated with the daily intake of soy‐based foods in Chinese women (Chen et al. 1999). Overall, there is a significant correlation between soy intake and urine isoflavone excretion, further marking their status as effective biomarkers (Mori et al. 2022; Soukup et al. 2023). Therefore, it is necessary to develop a new quick detection method for the accurate level of biomarkers of soybean isoflavones in human urine to assess dietary intake of soy and supplement the diet surveys.

As a polar substance, soybean isoflavone has a parent nucleus of 3‐benzopyrone and contains two benzene rings, so it has a strong ultraviolet absorption and can be qualitatively detected by ultraviolet spectrophotometry (Migues et al. 2022). According to the structural characteristics of soybean isoflavones molecules, it can be quantitatively detected by characteristic color development and fluorescence reaction (Li et al. 2024). Recent detection methods of the soybean isoflavones were quantitatively analyzed by high‐performance liquid chromatography (Aboushanab et al. 2022), fluorescence spectroscopy (Sakamoto et al. 2021), nuclear magnetic resonance (Yi et al. 2019), enzyme‐linked immunosorbent assay (Sakamoto et al. 2015), and near‐infrared spectroscopy (Amanah et al. 2022). However, the capillary electrophoresis is complicated and has poor reproducibility, and liquid chromatography and mass spectrometry technology have the disadvantages of expensive equipment and tedious sample pretreatment. The matrix of the human body fluid sample is complex (such as urea, inorganic salts, uric acid, creatinine, etc.), which will interfere with the detection of soybean isoflavones (Chen et al. 2021). However, the low levels of biomarkers and the complexity of biosamples make the analytical assay of several biomarkers a challenging issue. Because of the nanomolar range levels of typical biomarkers in urine as well as the complex matrix of biological media, adequate sample preparation methods should be used for quantification of biomarkers (Hamidi et al. 2018). Suitable sample preparation runs remain a vital part of the puzzle of diagnostic level. Enhancing the detection limit of bioanalytical methods starts during the sample preparation procedure (Hamidi 2022).

Consequently, in order to reduce the urine matrix interference, it is vital to enrich and purify the soybean isoflavones in the sample solution before the instrument analysis. Methods commonly available for separating isoflavones and their derivatives from physiological liquids include liquid–liquid extraction or solid‐phase extraction (SPE).

SPE is an ideal choice for sample preparation due to its convenient operation, high selectivity, and green environment resulting from the small amount of organic solvents. The adsorption effect of solid‐phase extraction technology often depends on the adsorption agent (Yu et al. 2021); commonly, adsorption materials are multi‐walled carbon nanotubes (Xu et al. 2021), molecularly imprinted polymer (Chmangui et al. 2021), magnetic nanoparticle (Tang et al. 2022), graphene material, etc. However, the separation ability of these materials is weak, and secondary pollution may be generated during the extraction process. Therefore, the development of new solid phase adsorption materials is one of the more difficult but popular directions in analytical science. Electrospinning nanofibers are a kind of solid‐phase extraction adsorption material that has attracted much attention recently, with the characteristics of large specific surface area, high porosity, and small pore depth (Jiang et al. 2019; Liang et al. 2020; Wan et al. 2022), which makes the adsorbent have better selectivity and improves the extraction enrichment efficiency. The study of new analytical methods on the basis of nanofiber solid‐phase extraction has attracted extensive attention from researchers. Compared with the traditional liquid phase extraction method, the extraction process of this method does not need to consume a lot of organic solvent, and the extraction column can be used repeatedly, which has the advantages of green environmental protection.

**FIGURE 1:**
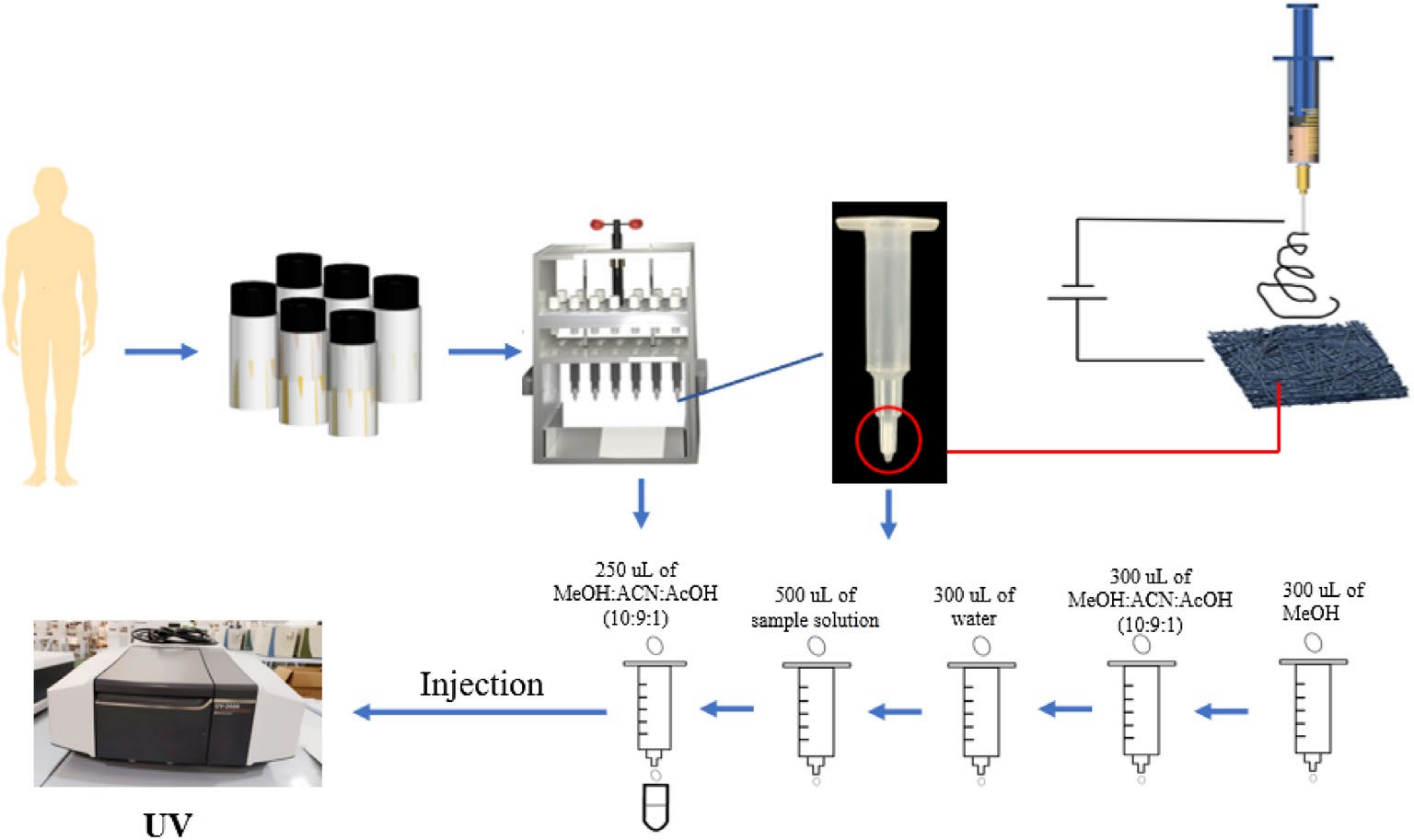
Schematic of isoflavones detection on the basis of packed‐fiber solid‐phase extraction.

This study prepares selective sample pretreatment materials by electrospinning technology as a sample treatment sorbent to enrich and purify soybean isoflavones in urine (as shown in Figure 1), and establish a highly selective quantitative analysis and rapid detection method for soybean isoflavones in urine on the basis of nanofiber‐packed solid‐phase extraction by using an ultraviolet spectrophotometer, expecting to significantly remove interfering substances, concentrate the target, improve the precision and accuracy of the detection, and develop a new quick detection method for the accurate level of biomarkers of soybean isoflavones in human urine.

## 2 Materials and Methods

### 2.1 Chemicals and Reagents

Soybean isoflavones (purity 98%) were purchased from Shanghai Yuanye Bio‐Technology Company. Sodium chloride, hydrochloric acid, methanol, and acetonitrile (chromatographic pure) were purchased from Shanghai Aladdin Company. Glacial acetic acid was purchased from Shanghai Maclin Company. Sodium dodecyl sulfate, N, N‐dimethylformamide (DMF), tetrahydrofuran (THF), polystyrene (PS, Mw = 192,000 g/mol), and polyvinylpyrrolidone (PVP, Mw = 58,000 g/mol) were purchased from Shanghai Sinopharm Group.

### 2.2 Instruments

Instruments for electrospinning are high voltage DC power supply and a syringe pump (Model HK‐400, Shenzhen, China). Instruments for determining the pH value are a pH meter with a composite electrode (Jiangsu, China). Instruments for nanofiber characterization are a Hitachi Regulus 8100 Scanning Electron Microscope (SEM, Hitachi, Japan), an FEI Tecnai F20 transmission electron microscope (TEM, Thermo Company, USA), and a VERTEX 80v Fourier Transform Infrared Spectrometer (Bruce GMBH, Germany). Instrument sample testing was UV–visible Spectrophotometer (Shimadzu Corporation, Japan).

### 2.3 Preparation of Nanofibers and SPE Extraction Device

The solution is configured as follows: 1 g PS is put into a mixture of 6 mL THF and 4 mL DMF (6:4, v/v) and dissolved at 20°C for 12 h to obtain the uniform polymer solution for electrospinning. The obtained 10% (w/v) PS solution is filled into a 10 mL syringe, and a 23‐gauge stainless steel needle is installed in the front section of the syringe and connected to the positive terminal of the high‐voltage power supply. The aluminum foil serving as the collection device is placed opposite the syringe and connected to the negative electrode of the high‐voltage power supply. Under the action of the electric field, the solution is sprayed out, the solvent is volatilized, and the fiber is formed and collected on the aluminum foil. Then, 10% PS‐5% PAN nanofibers, 10% PS‐5% AR nanofibers, 10% PS nanofibers, 10% PS‐5% PVP nanofibers, and 10% PS‐5% PES nanofibers were prepared respectively by the same way. The design and results of the optimization experiment on electrospinning parameters are shown in Table S1. According to the experimental results, the following electrospinning parameters are set as follows: the receiving distance from the tip of the needle to the collection screen is 15 cm, and 19 kV is applied; the flow rate of the spinning solution is 1.5 mL/h, and the whole electrospinning process is carried out at 25°C and 35% relative humidity. The direct view of the electrospinning nanofibers is shown in Figure S1. Then, the SPE columns are prepared by filling the bottom of an empty extraction column with nanofibers. The nanofibers in the column are lightly compacted with a 1 mm diameter wire to ensure efficient extraction.

### 2.4 Sample Pretreatment

The dry sample was crushed through a 100‐mesh sieve, weighed, and placed 1.0 g into a 25 mL colorimetric tube, added the extraction liquid methanol and water mixture (4:1, V:V), fixed the volume to the scale, shook well, underwent ultrasonic extraction for 30 min, left for 30 min, and obtained the supernatant. Filter the supernatant with a 0.22 μm organic phase filter membrane to be tested.

We recruited three men and three women living in Nanjing (China) as volunteers, aged between 20 and 25 years old, and collected their morning urine samples and stored them at −20°C for future use. All volunteers participated in the trial voluntarily. We thawed the sample to room temperature before testing it. After 30 min of ultrasound, the supernatant was centrifuged at 12,000 r/min for 15 min; then the supernatant after centrifuging was stored for the subsequent packed‐fiber solid‐phase extraction procedure.

### 2.5 Packed‐Nanofiber Solid‐Phase Extraction

The design of the pin‐shaped extraction column with a semi‐automatic SPE processor can be used for automatic batch processing of sample liquid, as shown in Figure S2. Prior to extraction, fibers were coated in solid‐phase extraction columns with 150 μL of methanol and 150 μL of water, respectively. The 500 μL sample is then transferred into the SPE column, where the column pressure is provided by a rotating pressure rod‐driven supercharger that pushes; the sample solution flows uniformly through the SPE column, and the flow rate is controlled to a drop every 4–5 s. As the solution passes through the SPE column, the target is captured by the filled adsorbent. After extraction, 250 μL of the eluent with methanol: acetonitrile: acetic acid (v:v:v, 50:45:5) was used to drip wash the adsorbent to obtain the eluant containing the target substance. Finally, the eluent collected was filtered through a 0.22 μm filter membrane and then analyzed with an ultraviolet spectrophotometer.

### 2.6 Method Validation

The standard solution of 0.05–30 μg/mL was prepared by adding soybean isoflavones into blank artificial urine. The standard curve was constructed with the level of the target substance as the horizontal coordinate and the corresponding light absorption value as the vertical coordinate. The limit of detection (LOD) and limit of quantification (LOQ) were defined with 3 and 10 times ratios of signal to noise, respectively. The recovery rate was evaluated by comparing the concentration of the eluent with the sample solution. The specific formula is as follows,

$$\text{Recovery}\;\left(\%\right)=\frac{C_{f}\times V_{f}}{C_{i}\times V_{i}}\times 100\%$$

where *C*_i_ and *V*_i_ are the concentration (μg/L) and volume of the sample solution, respectively. *C*_f_ and *V*_f_ are the concentration (μg/L) and volume of the eluent after SPE, respectively.

### 2.7 Evaluation of the Green Characteristics and Practicability

The method uses a small amount of organic solvent, has the characteristics of environmental friendliness, and requires a small sample size, convenient and fast. We used the following software to evaluate the green characteristics and practicability of the method: Analytical Greenness Metric for Sample Preparation (AGREEprep) (Wojnowski et al. 2022), the Complex Modified‐GAPI (ComplexMo‐GAPI) (Mansour et al. 2024), and the following software to evaluate the practicality of the method: the Blue Applicability Grade Index (BAGI) (Manousi et al. 2023).

## 3 Results and Discussion

### 3.1 Characterization of Nanofibers

The type and concentration of spinning solution, spinning voltage, receiving plate distance, ambient temperature, and humidity all affect the morphology and physical and chemical properties of the prepared nanofibers. Among them, the concentration and type of spinning solution have the most obvious influence (Kang et al. 2007). The usage of DMF as a solvent allows PS to fully dissolve and form a more uniform and stable fiber (Xin et al. 2024), and the low boiling point of THF makes the nanofibers have a microporous structure, which improves the porosity of the fibers (Y. Z. Wang, Hou, et al. 2023); therefore, the mixture of THF and DMF was chosen as the spinning solution solvent. The PS molecule is an irregular molecule with a saturated carbon main chain; the side groups are conjugated benzene rings, and this structural feature gives it a good supporting force (Sharma and Garg 2021). The beaded structure on nanofibers contains more nanopores and a larger specific surface area, but a high concentration of PS will gradually reduce the number of beaded structures, thus affecting the adsorption efficiency (Nygaard et al. 2011). However, the higher the concentration of PS, the larger the diameter of the formed fiber, which affects the specific surface area of the fiber (Kwak et al. 2021). Therefore, 10% PS nanofibers were selected to probe the effect on the adsorption of targets.

**FIGURE 2:**
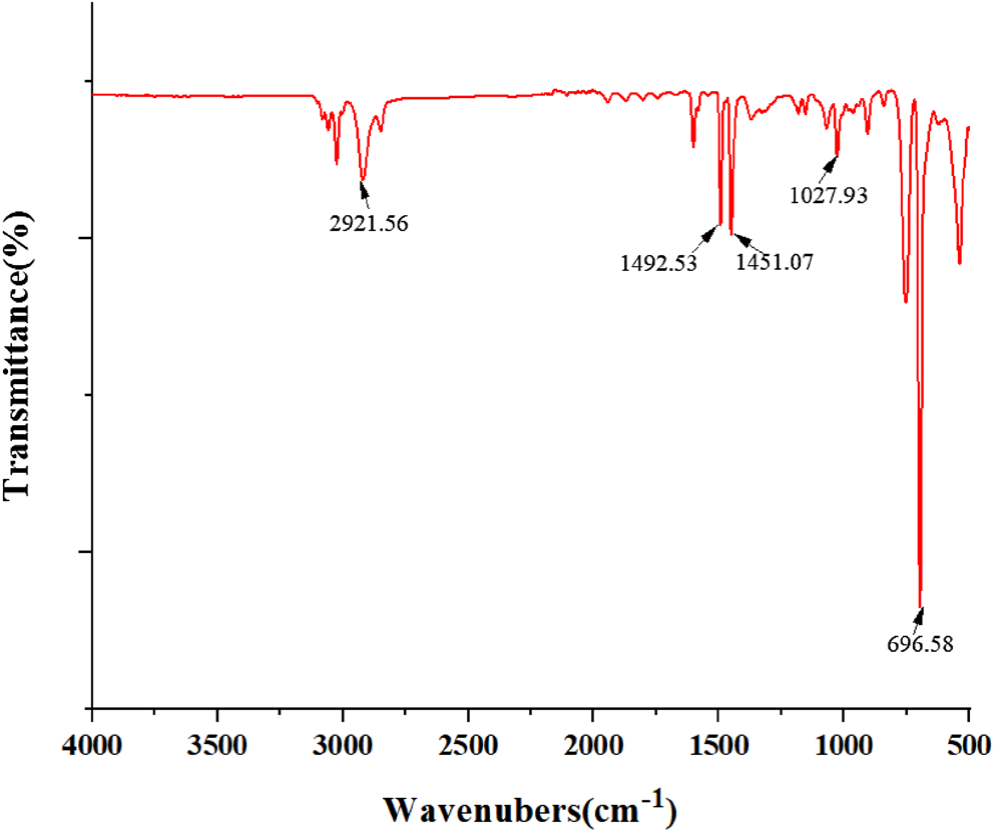
Infrared spectra of 10% PS nanofibers.

According to the literature, the infrared spectrogram of PS nanofibers has a styrene ring formation peak at 696.58 cm^−1^. The two bands 1492.53 and 1451.07 cm^−1^ belong to the C=C vibration of the aromatic ring. The other band at 1027.93 cm^−1^ corresponds to the vinyl C—H bond. Finally, the 2921.56 cm^−1^ band is generated by the C—H bond with spin SP_2_. As Figure 2 shows that the peak position is consistent with the literature, it can be considered that we have prepared excellent PS nanofibers (Amer et al. 2024).

**FIGURE 3:**
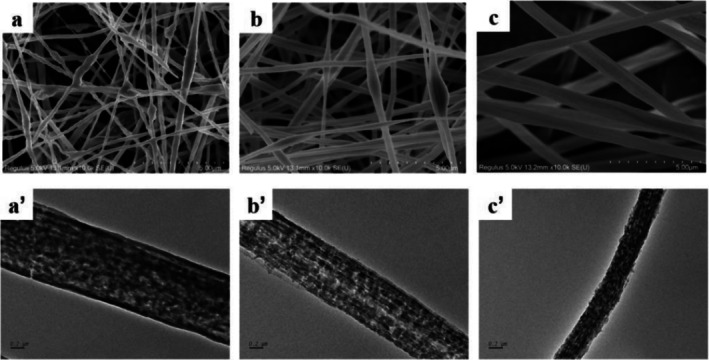
SEM and TEM images of (a and a') 10% PS nanofibers, (b and b') 5% PS‐5% AR nanofibers, and (c and c') 5% PS‐5 PVP% nanofibers.

The surface properties of nanofiber materials were studied by SEM and TEM, and the SEM images show that 10% PS nanofibers present a dense network structure with a few beads attached, as shown in Figure 3. Compared with 5% PS‐5% PVP, 5% PS‐5% AR, and 10% PS fibers are finer, which means that they have a larger specific surface area and better adsorption effect.

### 3.2 Determination of the Detection Wavelength of Isoflavones

The high, medium, and low concentrations of soybean isoflavones (0.05, 1, 2 μg/mL) were determined, and according to the ultraviolet spectrogram (shown as Figure S3), isoflavones had the maximum absorption peak at 260 nm, which was consistent with the literature reported.

### 3.3 Optimization of Solid‐Phase Extraction Conditions

#### 3.3.1 Type of Nanofibers

Different types of nanofibers have different functional groups and microstructures, which affect their interaction with isoflavones and lead to different adsorption effects (Lhotská et al. 2024). In this project, the types of extractant electrospinning nanofibers in sample pretreatment were optimized, as shown in Figure S4. However, since the combination of eluent and PES has a high light absorption value at 260 nm, which will affect the detection of target objects, we finally chose 10% PS as the optimal fiber.

#### 3.3.2 Type and Concentration of Salt Ion

Adding salt ions to the sample solution will produce ion effects and affect the adsorption efficiency of the sample. This project optimized the type and concentration of salt ions added to the sample solution in sample pre‐treatment and finally selected 1.25 × 10^−3^ g/mL SDS as the best extraction condition, as shown in Figure S5. This may be because polystyrene, the backbone of the nanofiber, is a non‐polar polymer that can bind to the hydrophobic tail of sodium lauryl sulfate, resulting in an increased ratio of hydrophilic heads, improving the adsorption properties of the nanofibers.

#### 3.3.3 Type of Eluent

The type of eluent is an important factor in the optimization of SPE, and it has a significant effect on the desorption efficiency of the target in nanofibers. Therefore, the type of eluent used for sample pretreatment was optimized, which included methanol (100%), methanol:water (v:v, 6:4), acetonitrile (100%), methanol:water: 0.1% phosphoric acid (v:v:v, 4:4:2), and methanol:acetonitrile:acetic acid (v:v:v, 50:45:5). Finally, the eluent of methanol: acetonitrile: acetic acid (v:v:v, 50:45:5) was selected as the optimal eluent type, as shown in Figure S6.

#### 3.3.4 Dosage of Nanofibers

Increasing the dosage of nanofibers will increase their specific surface area and porosity, so as to improve its adsorption effect. In this experiment, the amount of fiber was optimized to obtain a suitable recovery rate. Finally, considering the recovery rate and cost, 12 mg was selected as the optimal filling amount, as shown in Figure S7.

#### 3.3.5 Effect of Extraction Time

The adsorbent needs enough extraction time to adsorb the complete target material to achieve extraction equilibrium (Deji et al. 2022). Therefore, we obtain the best extraction time by exploring the extraction effect of different extraction times. As Figure S8 shows, the extraction efficiency gradually increased with the increase of the time for sample solution flowing through the solid‐phase extraction column. When the extraction time was 7 min, the adsorption efficiency of the nanofibers to the soybean isoflavones object reached the highest value. Therefore, 7 min was selected as the optimal extraction time.

### 3.4 Method Validation

The standard stock solution of soybean isoflavone was diluted with artificial urine, and the calibration curve was obtained in the level range of 0.05–30 μg/mL. The standard curve of soybean isoflavone was linear in the specified level range (*R* ^2^ ≥ 0.99), and the regression equation was *y* = 0.0576*x* + 0.0479; the LOD is 0.01 μg/mL.

The recovery rate was determined with three different concentrations (50, 75, 125 ng/mL) of labeled blank artificial urine samples. This method has a good effect on sample extraction; in the samples with different concentrations, the recoveries were 70.5%–82.5%, which were all within the acceptable range.

**FIGURE 4:**
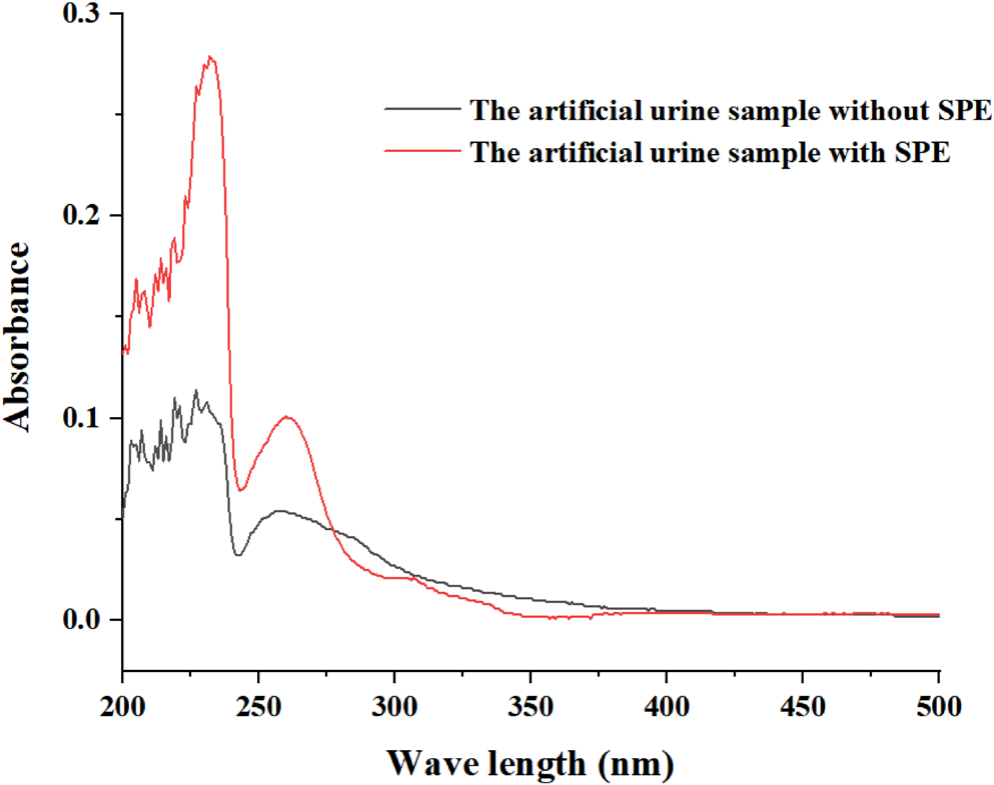
Ultraviolet spectra of the labeled samples.

We also observed artificial urine after SPE procedures. As shown in Figure 4, the interference of the impurity peak with the target peak is significantly reduced, and the target peak is obvious and prominent, which is conducive to the detection of isoflavones in complex substrates. The experiments show that SPE has a good purification effect on impurities and an obvious adsorption and enrichment effect on target material.

Recently, many emerging sample pretreatment methods have been applied to extract and detect isoflavones from biological fluid samples. The comparison of selected analytical parameters of the proposed method with reported methods is studied to show how this method advances over the existing one and impacts its applicability. As shown in Table 1, by comparing the key analytical parameters between the reported and proposed methods, our method also showed attractive feasibility and acceptability, providing similar detection limits, less organic solvent usage, and extremely short detection time with reasonable recovery.

**TABLE 1:** Comparison of selected analytical parameters of the proposed method with reported methods.

Matrix	Method of sample pretreatment	Detection methods	Recovery (%)	LOD	Detection time	References
Breast milk	Liquid–liquid extraction	LC–MS/MS	81.99–119	0.0187 nmol/mL	5 min	Min et al. (2020)
Human urine	Liquid–liquid extraction	RP‐HPLC	89.3–99.0	12.9 nM	29 min	Wang et al. (2006)
Bovine plasma and milk	Solid‐phase extraction (Oasis HLB Column)	HPLC‐MS/MS	82.0–112.0	5 ng/mL	16 min	Krajcová et al. (2010)
Human urine	Solid‐phase extraction (Hypersil GOLD Column)	UHPLC	≥ 70.0	12.8 ng/mL	4.5 min	Baranowska et al. (2011)
Human urine	Solid‐phase extraction (Bond Elut C18)	UHPLC	70.11–102.94	2.93 nM	22 min	Redruello et al. (2015)
Human urine	Methanol extraction	LC‐ESI‐MS/MS	—	—	> 70 min	Chang et al. (2009)
Human urine	Solid‐phase extraction (PS nanofibers)	UV–Vis	70.5–82.5	10 ng/mL	0.5 min	This paper

### 3.5 Matrix Effect

To investigate matrix effects in urine samples, standard solutions were diluted to 10, 20, and 50 ng/mL with methanol and blank artificial urine, respectively. The matrix effect was evaluated by comparing the slope of the matrix calibration curve with the slope of the solvent calibration curve (J. Li, Twaddle, et al. 2023). The calculation formula is as follows.

$$\mathrm{ME}=\left({\text{Slope}}_{1}/{\text{Slope}}_{2}-1\right)\times 100\%$$

where Slope_1_ is the slope of the matrix calibration curve, and Slope_2_ is the slope of the solvent calibration curve. The results showed that the matrix effect of isoflavones in artificial urine samples was 13.9%. The data are less than 15%, indicating that the influence of the urine matrix was well‐controlled.

### 3.6 Actual Sample Testing

#### 3.6.1 Detection of Isoflavones in Food

In order to verify the feasibility and application value of the pre‐treatment method developed, we selected six kinds of food (soya bean, mung bean, ormosia bean, black bean, health care products, and bean curd) as actual samples and used the established analytical method to detect the content of soybean isoflavones. As can be seen from Figure S9, soya bean has the highest content of soybean isoflavones and is the most important source of isoflavones intake.

#### 3.6.2 Analysis of Isoflavones in Actual Urine Samples

The applicability of the PS nanofiber ultraviolet spectrophotometer was investigated by analyzing six actual urine samples (three male and three female), as shown in Table 2. No isoflavones were detected in three male samples. Soy isoflavones were detected in only one female sample. In summary, the PS nanofiber membrane can adsorb soybean isoflavones in human urine samples, showing great potential in the pretreatment of a large number of human samples.

**TABLE 2:** Detection results of isoflavones in actual urine samples.

Sample	Detection (μg/mL)	Sample	Detection (μg/mL)
Male 1	Nd	Female 1	Nd
Male 2	Nd	Female 2	0.258
Male 3	Nd	Female 3	Nd

Abbreviation: Nd, not detected.

### 3.7 Evaluation of the Green Characteristics and Practicability

**FIGURE 5:**
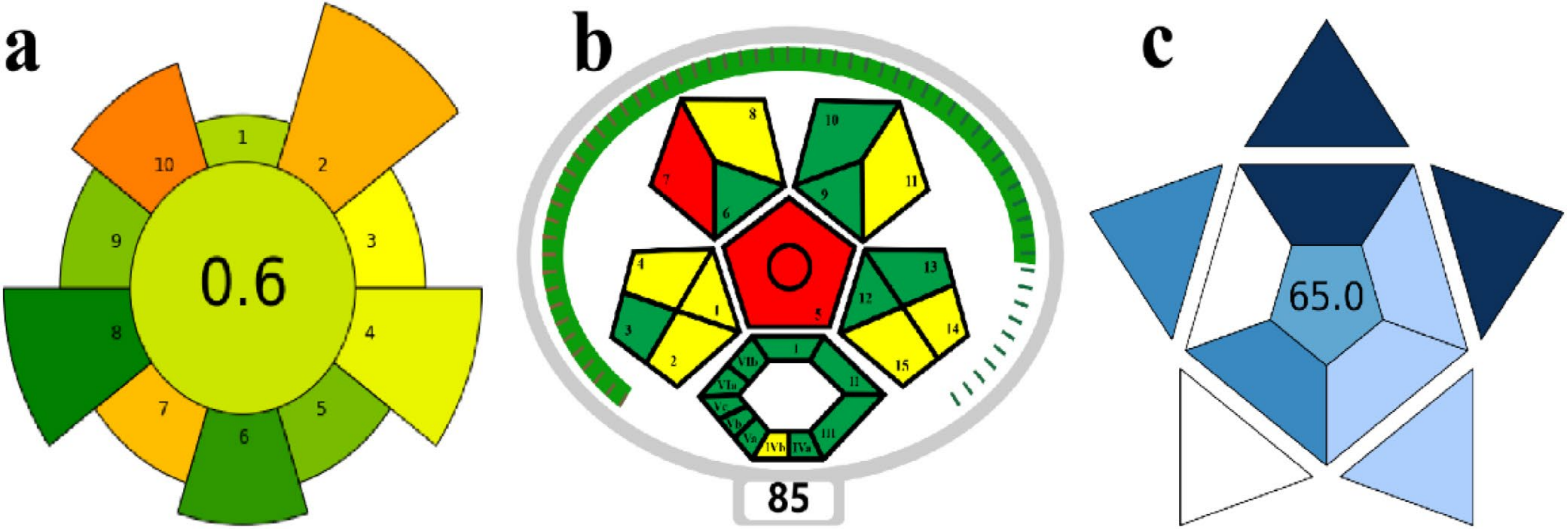
(a) AGREEprep, (b) BAGI, and (c) ComplexMo‐GAPI pictograms of the validated method.

The green sample preparation process is now highly valued, so we are using AGREEprep, ComplexMo‐GAPI, and BAGI metrics to evaluate the environmental friendliness and safety of validated methods. This method is performed without changing the weight of the original standard. The pictogram of the validation method is shown in Figure 5, and the specific evaluation indicators are shown in Tables S2–S4.

We firstly selected AGREEprep to evaluate the Green Characteristics of this method (Figure 5a). The sample preparation was performed in an online/in situ mode and was divided into three steps: activation, extraction, and elution. The waste volume generated for each sample was 12 mg nanofibers and 1.4 mL organic solvent. The sample volume was 0.5 mL, and approximately 48 samples could be prepared in 1 h. The entire extraction process was performed using a homemade extraction column combined with a semi‐automated extraction device. The entire system required neither heating nor cooling, so it can be assumed that the energy demand for each prepared sample is low. The method received a final score of 0.6. It should be noted that on‐site sample preparation and semi‐automation, as well as the small amount of sample required, are the advantages of this procedure.

We chose to use ComplexMo‐GAPI to conduct a complete evaluation of the entire method (Figure 5b). The key aspects are similar to those emphasized by other indicators (sample preparation efficiency, use of organic solvents, safety evaluation, etc.); the final score of this method is 85 points. This method performs well in preparation efficiency, but the use of organic reagents such as methanol and acetonitrile will cause certain waste generation. However, the volume of organic reagents used in this method is very small, which greatly reduces the pollution caused by organic reagents.

Finally, we selected BAGI to evaluate the applicability of this method (Figure 5c). This method uses common commercially available reagents and simple‐to‐operate instruments to perform qualitative and quantitative analysis of a single analyte. This method can process 48 samples in 1 h. The required sample volume is small, only 0.5 mL, but it needs to be pre‐concentrated by solid‐phase extraction to achieve the required sensitivity. The entire operation is performed in semi‐automated mode. Therefore, the BAGI score of this method is 65, which proves its superiority in practicality and applicability.

## 4 Conclusion

In this study, the electrospun nanofibers were utilized as a solid‐phase extraction adsorbent to purify human urine samples and successfully established an analytical method on the basis of packed‐fiber solid‐phase extraction and UV detection. Five types of nanofibers were prepared by the electrospinning method, and a home‐made extraction device was prepared, too. The factors of fiber‐filled dosage, type and concentration of salt ion, and type of eluent were optimized. Under the optimal conditions, the sample pretreatment displayed a preferable effect on the removal of impurities and the enrichment of target analytes, and the method can notably remove the interference of matrix effect and remarkably purify and enrich the target analytes. The new detection method for the biomarkers of soybean isoflavones in human urine on the basis of packed‐nanofiber solid‐phase extraction is of simple operation, less organic solvents, and more environmentally friendly, economical, and suitable for the quantitative determination of soy isoflavones in complex matrix samples.

## Author Contributions

Lanling Chu: writing – original draft, writing – review and editing, and methodology. Yuqi Dai: writing – original draft, formal analysis, and data curation. Qinghai Hu: methodology, validation, and data curation. Erzheng Su: supervision, and writing – review and editing. Shuo Qi: visualization and writing – review and editing. Xiaoman Jiang: project administration, writing – review and editing, and funding acquisition. Anni Fu: formal analysis and data curation. Qianqian Jiang: resources, methodology, and funding acquisition. Xuejun Kang: conceptualization, methodology, writing – review, and project administration.

## Ethics Statement

The authors have nothing to report.

## Consent

The authors have nothing to report.

## Supporting information


**Data S1:** fsn371116‐sup‐0001‐TableS1‐S4‐FigureS1‐S9.docx.

## Data Availability

The authors have nothing to report.

## References

[fsn371116-bib-0001] Aboushanab, S. A. , V. A. Shevyrin , G. P. Slesarev , et al. 2022. “Antioxidant and Cytotoxic Activities of Kudzu Roots and Soy Molasses Against Pediatric Tumors and Phytochemical Analysis of Isoflavones Using HPLC‐DAD‐ESI‐HRMS.” Plants‐Basel 11: 741. 10.3390/plants11060741. 35336625 PMC8955742

[fsn371116-bib-0002] Adlercreutz, H. , H. Honjo , A. Higashi , et al. 1991. “Urinary Excretion of Lignans and Isoflavonoid Phytoestrogens in Japanese Men and Women Consuming a Traditional Japanese Diet.” American Journal of Clinical Nutrition 54, no. 6: 1093–1100. 10.1093/ajcn/54.6.1093. 1659780

[fsn371116-bib-0003] Amanah, H. Z. , S. S. Tunny , R. E. Masithoh , et al. 2022. “Nondestructive Prediction of Isoflavones and Oligosaccharides in Intact Soybean Seed Using Fourier Transform Near‐Infrared (FT‐NIR) and Fourier Transform Infrared (FT‐IR) Spectroscopic Techniques.” Food 11, no. 2: 232. 10.3390/foods11020232. PMC8774574 35053964

[fsn371116-bib-0004] Amer, A. M. , S. I. El‐Dek , A. A. Farghali , and N. Shehata . 2024. “Management of Ibuprofen in Wastewater Using Electrospun Nanofibers Developed From PET and PS Wastes.” Chemosphere 359: 142313. 10.1016/j.chemosphere.2024.142313. 38735499

[fsn371116-bib-0005] Baranowska, I. , S. Magiera , and J. Baranowski . 2011. “UHPLC Method for the Simultaneous Determination of β‐Blockers, Isoflavones, and Flavonoids in Human Urine.” Journal of Chromatographic Science 49, no. 10: 764–773. 10.1093/chrsci/49.10.764. 22080804

[fsn371116-bib-0006] Chang, H. C. , J. Han , and R. L. Prior . 2009. “Detection of Conjugated Soy Metabolites in Urinary and Tissue Samples After Methanol Extraction.” Journal of Food and Drug Analysis 17: 43–51. 10.38212/2224-6614.2307.

[fsn371116-bib-0007] Chen, Y. , T. Li , H. L. Ji , et al. 2021. “Associations of Maternal Soy Product Consumption and Urinary Isoflavone Concentrations With Neonatal Anthropometry: A Prospective Cohort Study.” Environmental Pollution 274: 115752. 10.1016/j.envpol.2020.115752. 33190984

[fsn371116-bib-0008] Chen, Z. , W. Zheng , L. J. Custer , et al. 1999. “Usual Dietary Consumption of Soy Foods and Its Correlation With the Excretion Rate of Isoflavonoids in Overnight Urine Samples Among Chinese Women in Shanghai.” Nutrition and Cancer 33, no. 1: 82–87. 10.1080/01635589909514752. 10227048

[fsn371116-bib-0009] Chmangui, A. , G. Jayasinghe , M. R. Driss , et al. 2021. “Assessment of Trace Levels of Aflatoxins AFB_1_ and AFB_2_ in Non‐Dairy Beverages by Molecularly Imprinted Polymer Based Micro Solid‐Phase Extraction and Liquid Chromatography‐Tandem Mass Spectrometry.” Analytical Methods 13, no. 30: 3433–3443. 10.1039/d1ay00793a. 34259236

[fsn371116-bib-0010] Deji, Z. M. , X. Zhang , P. Liu , X. Wang , K. Abulaiti , and Z. Z. Huang . 2022. “Electrospun UiO‐66‐F4/Polyacrylonitrile Nanofibers for Efficient Extraction of Perfluoroalkyl and Polyfluoroalkyl Substances in Environmental Media.” Journal of Hazardous Materials 430: 128494. 10.1016/j.jhazmat.2022.128494. 35739675

[fsn371116-bib-0011] Duan, Y. , Q. Qi , Z. H. Liu , M. Zhang , and H. Q. Liu . 2022. “Soy Consumption and Serum Uric Acid Levels: A Systematic Review and Meta‐Analysis.” Frontiers in Nutrition 9: 975718. 10.3389/fnut.2022.975718. 36118757 PMC9479323

[fsn371116-bib-0012] Geng, Y. J. , S. L. Chen , Y. Yang , et al. 2022. “Long‐Term Exposure to Genistein Inhibits the Proliferation of Gallbladder Cancer by Downregulating the MCM Complex.” Science Bulletin 67, no. 8: 813–824. 10.1016/j.scib.2022.01.011. 36546234

[fsn371116-bib-0013] Goja, A. , S. Al‐Otaishan , L. Al‐Awami , et al. 2023. “Effect of Soy Isoflavones on Bone Health Among Female University Students: A Pilot Study.” Emirates Journal of Food and Agriculture 35, no. 8: 722–730. 10.9755/ejfa.2023.3131.

[fsn371116-bib-0014] Gormley, I. C. , Y. X. Bai , and L. Brennan . 2020. “Combining Biomarker and Self‐Reported Dietary Intake Data: A Review of the State of the Art and an Exposition of Concepts.” Statistical Methods in Medical Research 29, no. 2: 617–635. 10.1177/0962280219837698. 30943855

[fsn371116-bib-0015] Hamidi, S. , N. Alipour‐Ghorbani , and A. Hamidi . 2018. “Solid Phase Microextraction Techniques in Determination of Biomarkers.” Critical Reviews in Analytical Chemistry 48: 239–251. 10.1080/10408347.2017.1396885. 29337594

[fsn371116-bib-0016] Hamidi, S. 2022. “Recent Advances in Solid‐Phase Extraction as a Platform for Sample Preparation in Biomarker Assay.” Critical Reviews in Analytical Chemistry 53: 199–210. 10.1080/10408347.2021.1947771. 35192409

[fsn371116-bib-0017] Jang, H. H. , Y. M. Lee , J. S. Choe , and O. Kwon . 2021. “Validation of Soy Isoflavone Intake and Its Health Effects: A Review of the Development of Exposure Biomarkers.” Nutrition Research and Practice 15, no. 1: 1–11. 10.4162/nrp.2021.15.1.1. 33542788 PMC7838478

[fsn371116-bib-0018] Jiang, N. G. , M. Zhao , S. H. Lang , et al. 2019. “High‐Throughput and High‐Efficient Micro‐Solid Phase Extraction Based on Sulfonated‐Polyaniline/Polyacrylonitrile Nanofiber Mats for Determination of Fluoroquinolones in Animal‐Origin Foods.” Journal of Agricultural and Food Chemistry 67, no. 24: 6892–6901. 10.1021/acs.jafc.9b01312. 31125221

[fsn371116-bib-0019] Kang, X. J. , C. Pan , Q. Xu , et al. 2007. “The Investigation of Electrospun Polymer Nanofibers as a Solid‐Phase Extraction Sorbent for the Determination of Trazodone in Human Plasma.” Analytica Chimica Acta 587, no. 1: 75–81. 10.1016/j.aca.2007.01.021. 17386756

[fsn371116-bib-0020] Krajcová, A. , V. Schulzová , J. Lojza , L. Krizová , and J. Hajslová . 2010. “Phytoestrogens in Bovine Plasma and Milk ‐ LC‐MS/MS Analysis.” Czech Journal of Food Sciences 28, no. 4: 264–274. 10.17221/138/2010-CJFS.

[fsn371116-bib-0021] Kwak, B. E. , H. J. Yoo , E. Lee , and D. Kim . 2021. “Large‐Scale Centrifugal Multispinning Production of Polymer Micro‐ and Nanofibers for Mask Filter Application With a Potential of Cospinning Mixed Multicomponent Fibers.” ACS Macro Letters 10, no. 3: 382–388. 10.1021/acsmacrolett.0c00829. 34192093

[fsn371116-bib-0022] Lei, L. F. , S. C. Hui , Y. S. Chen , H. J. Yan , J. Yang , and S. W. Tong . 2024. “Effect of Soy Isoflavone Supplementation on Blood Pressure: A Meta‐Analysis of Randomized Controlled Trials.” Nutrition Journal 23: 53. 10.1186/s12937-024-00941-5. 38760764 PMC11102154

[fsn371116-bib-0023] Lhotská, I. , A. Kholová , F. Svec , and D. Satínsky . 2024. “Nanofibers Prepared From Synthetic Polymers and Biopolymers as Advanced Extraction Materials for Sample Preparation Prior to Liquid Chromatography.” Trac‐Trends in Analytical Chemistry 180: 117912. 10.1016/j.trac.2024.117912.

[fsn371116-bib-0025] Li, W. , N. C. Twaddle , B. Spray , B. Nounamo , B. Monzavi‐Karbassi , and R. Hakkak . 2023. “Feeding Soy Protein Concentrates With Low and High Isoflavones Alters 9 and 18 Weeks Serum Isoflavones and Inflammatory Protein Levels in Lean and Obese Zucker Rats.” Journal of Medicinal Food 26, no. 2: 120–127. 10.1089/jmf.2022.0100. 36720082

[fsn371116-bib-0026] Li, W. , X. P. Zhang , S. L. Wang , X. F. Gao , and X. L. Zhang . 2024. “Research Progress on Extraction and Detection Technologies of Flavonoid Compounds in Foods.” Food 13, no. 4: 628. 10.3390/foods13040628. PMC10887530 38397605

[fsn371116-bib-0027] Liang, S. H. , N. G. Jian , J. K. Cao , et al. 2020. “Rapid, Simple and Green Solid Phase Extraction Based on Polyaniline Nanofibers‐Mat for Detecting Non‐Steroidal Anti‐Inflammatory Drug Residues in Animal‐Origin Food.” Food Chemistry 328: 127097. 10.1016/j.foodchem.2020.127097. 32470774

[fsn371116-bib-0028] Manousi, N. , W. Wojnowski , J. Plotka‐Wasylka , and V. Samanidou . 2023. “Blue Applicability Grade Index (BAGI) and Software: A New Tool for the Evaluation of Method Practicality.” Green Chemistry 25, no. 19: 7598–7604. 10.1039/d3gc02347h.

[fsn371116-bib-0029] Mansour, F. R. , K. M. Omer , and J. Płotka‐Wasylka . 2024. “A Total Scoring System and Software for Complex Modified GAPI (ComplexMoGAPI) Application in the Assessment of Method Greenness.” Green Analytical Chemistry 10: 100126. 10.1016/j.greeac.2024.100126.

[fsn371116-bib-0030] Migues, V. H. , J. M. David , A. F. Gomes , and J. P. David . 2022. “Determination of Soybean Isoflavone by HPLC/DAD and Simple UV Spectroscopic Analysis: A Comparative Study.” Food Analytical Methods 15, no. 2: 367–376. 10.1007/s12161-021-02120-2.

[fsn371116-bib-0031] Min, J. , Z. Wang , C. Liang , et al. 2020. “Detection of Phytoestrogen Metabolites in Breastfed Infants' Urine and the Corresponding Breast Milk by Liquid Chromatography–Tandem Mass Spectrometry.” Journal of Agricultural and Food Chemistry 68: 3485–3494. 10.1021/acs.jafc.9b08107. 32093471

[fsn371116-bib-0032] Mori, M. , M. Sagara , H. Mori , and Y. Yamori . 2022. “Grading of Japanese Diet Intakes by 24‐Hour Urine Analysis of Taurine and Soy Isoflavones in Relation to Cardiovascular Risks.” Advances in Experimental Medicine and Biology 1370: 173–184. 10.1007/978-3-030-93337-1_17. 35882793

[fsn371116-bib-0033] Naghshi, S. , H. Tutunchi , M. Yousefi , et al. 2024. “Soy Isoflavone Intake and Risk of Cardiovascular Disease in Adults: A Systematic Review and Dose‐Response Meta‐Analysis of Prospective Cohort Studies.” Critical Reviews in Food Science and Nutrition 64, no. 18: 6087–6101. 10.1080/10408398.2022.2163372. 36705465

[fsn371116-bib-0034] Nygaard, J. V. , T. Uyar , M. L. Chen , P. Cloetens , P. Kingshott , and F. Besenbacher . 2011. “Characterisation of Internal Morphologies in Electrospun Fibers by X‐Ray Tomographic Microscopy.” Nanoscale 3: 3594–3597. 10.1039/c1nr10304k. 21842086

[fsn371116-bib-0035] Redruello, B. , L. Guadamuro , I. Cuesta , J. R. Alvarez‐Buylla , B. Mayo , and S. Delgado . 2015. “A Novel UHPLC Method for the Rapid and Simultaneous Determination of Daidzein, Genistein and Equol in Human Urine.” Journal of Chromatography. B, Analytical Technologies in the Biomedical and Life Sciences 1005: 1–8. 10.1016/j.jchromb.2015.09.029. 26444491

[fsn371116-bib-0036] Sakamoto, S. , H. Uchiyama , G. Yusakul , et al. 2021. “Open Sandwich Fluorescence‐Linked Immunosorbent Assay for Detection of Soy Isoflavone Glycosides.” Food Chemistry 361: 129829. 10.1016/j.foodchem.2021.129829. 34087571

[fsn371116-bib-0037] Sakamoto, S. , G. Yusakul , B. Pongkitwitoon , M. K. Paudel , H. Tanaka , and S. Morimoto . 2015. “Simultaneous Determination of Soy Isoflavone Glycosides, Daidzin and Genistin by Monoclonal Antibody‐Based Highly Sensitive Indirect Competitive Enzyme‐Linked Immunosorbent Assay.” Food Chemistry 169: 127–133. 10.1016/j.foodchem.2014.08.004. 25236207

[fsn371116-bib-0038] Sharma, T. , and M. Garg . 2021. “Pristine, Irradiated and Nanocomposite Polystyrene: Recent Experimental and Theoretical Developments.” Transactions on Electrical and Electronic Materials 22, no. 4: 394–418. 10.1007/s42341-021-00342-z.

[fsn371116-bib-0039] Soukup, S. T. , A. K. Engelbert , B. Watzl , A. Bub , and S. E. Kulling . 2023. “Microbial Metabolism of the Soy Isoflavones Daidzein and Genistein in Postmenopausal Women: Human Intervention Study Reveals New Metabotypes.” Nutrients 15, no. 10: 2352. 10.3390/nu15102352. 37242235 PMC10223177

[fsn371116-bib-0040] Sun, C. Y. , B. Guo , X. Liu , X. Xiao , X. Zhao , and C. C. China Multi‐Ethnic Cohort . 2022. “Interviewer Error Within the Face‐to‐Face Food Frequency Questionnaire in Large Multisite Epidemiologic Studies.” American Journal of Epidemiology 191: 921–929. 10.1093/aje/kwac024. 35136900 PMC9071521

[fsn371116-bib-0041] Tang, Z. T. , Q. R. Han , G. Yu , F. Liu , Y. Z. Tan , and C. Peng . 2022. “Fe_3_O_4_@PDA/MIL‐101(Cr) as Magnetic Solid‐Phase Extraction Sorbent for Mycotoxins in Licorice Prior to Ultrahigh‐Performance Liquid Chromatography‐Tandem Mass Spectrometry Analysis.” Food Science & Nutrition 10, no. 7: 2224–2235. 10.1002/fsn3.2832. 35844918 PMC9281945

[fsn371116-bib-0042] Wan, X. R. , H. R. Dai , H. Y. Zhang , H. Yang , F. Li , and Q. Xu . 2022. “Emerald‐Based Polyaniline‐Modified Polyacrylonitrile Nanofiber Mats Based Solid‐Phase Extraction for Efficient and Simple Detection of Sudan Dyes in Poultry Feed.” Microchemical Journal 181: 107824. 10.1016/j.microc.2022.107824.

[fsn371116-bib-0043] Wang, J. , L. Han , L. Zhu , and Y. Gao . 2006. “Determination of Isoflavones in Human Urine by High Performance Liquid Chromatograph.” Chinese Journal of Analytical Chemistry 34, no. 4: 569–572. 10.1016/S1872-2040(06)60030-3.

[fsn371116-bib-0044] Wang, X. B. , Y. L. Zhang , X. Y. Zhou , et al. 2023. “Soy Isoflavone Reduces LPS‐Induced Acute Lung Injury via Increasing Aquaporin 1 and Aquaporin 5 in Rats.” Open Life Sciences 18: 20220560. 10.1515/biol-2022-0560. 36820212 PMC9938540

[fsn371116-bib-0045] Wang, Y. Z. , C. Hou , Y. Q. Dai , et al. 2023. “Determination of Aflatoxin B1 by Novel Nanofiber‐Packed Solid‐Phase Extraction Coupled With a High Performance Liquid Chromatography‐Fluorescence Detector.” Analytical Methods 15, no. 4: 472–481. 10.1039/d2ay01753a. 36602291

[fsn371116-bib-0046] Willis, N. D. , A. J. Lloyd , L. Xie , et al. 2020. “Design and Characterisation of a Randomized Food Intervention That Mimics Exposure to a Typical UK Diet to Provide Urine Samples for Identification and Validation of Metabolite Biomarkers of Food Intake.” Frontiers in Nutrition 7: 561010. 10.3389/fnut.2020.561010. 33195362 PMC7609501

[fsn371116-bib-0047] Wojnowski, W. , M. Tobiszewski , F. Pena‐Pereira , and E. Psillakis . 2022. “AGREEprep ‐ Analytical Greenness Metric for Sample Preparation.” Trac‐Trends in Analytical Chemistry 149: 116553. 10.1016/j.trac.2022.116553.

[fsn371116-bib-0048] Xin, Q. P. , H. J. Gao , K. An , X. L. Ding , Y. Z. Zhang , and K. Y. Zhao . 2024. “High‐Performance Electrospun Polystyrene‐Based Nanofiber Membrane for Efficient SO_2_ Capture.” Separation and Purification Technology 330: 125411. 10.1016/j.seppur.2023.125411.

[fsn371116-bib-0049] Xu, H. W. , J. D. Sun , H. M. Wang , Y. Z. Zhang , and X. L. Sun . 2021. “Adsorption of Aflatoxins and Ochratoxins in Edible Vegetable Oils With Dopamine‐Coated Magnetic Multi‐Walled Carbon Nanotubes.” Food Chemistry 365: 130409. 10.1016/j.foodchem.2021.130409. 34256225

[fsn371116-bib-0050] Yi, Y. , B. Adrjan , J. Li , B. Hu , and S. Roszak . 2019. “NMR Studies of Daidzein and Puerarin: Active Anti‐Oxidants in Traditional Chinese Medicine.” Journal of Molecular Modeling 25, no. 7: 202. 10.1007/s00894-019-4090-8. 31243583

[fsn371116-bib-0051] Yu, J. , S. Y. Di , H. Yu , T. Ning , H. C. Yang , and S. K. Zhu . 2021. “Insights Into the Structure‐Performance Relationships of Extraction Materials in Sample Preparation for Chromatography.” Journal of Chromatography A 1637: 461822. 10.1016/j.chroma.2020.461822. 33360779

