# Supplementary material for: A New Detection Method for the Biomarkers of Soybean Isoflavones in Human Urine on the Basis of Packed‐Fiber Solid‐Phase Extraction
Source: Food Sci Nutr. 2025 Oct 30;13(11):e71116. doi: 10.1002/fsn3.71116 (PMC12573273; doi:10.1002/fsn3.71116)
Supplement: Supplementary file 1 — Data S1: fsn371116‐sup‐0001‐TableS1‐S4‐FigureS1‐S9.docx. [file FSN3-13-e71116-s001.docx]

**Supporting Information**

***for***

**A new detection method for the biomarkers of soybean isoflavones in human urine based on packed-nanofiber solid-phase extraction**

Lanling Chu^1^, Yuqi Dai^1^, Qinghai Hu^1, 3^, Erzheng Su^1^, Shuo Qi^1^, Xiaoman Jiang^2^, Anni Fu^2^, Qianqian Jiang^2*^, Xuejun Kang^3*^

^1^National Key Laboratory for the Development and Utilization of Forest Food Resources, Nanjing Forestry University, Nanjing 210037, PR China.

^2^Yantai Key Laboratory of Special Medical Food (Preparatory), School of Food and Biological Engineering, Yantai Institute of Technology, Yantai 264005, PR China.

^3^School of Biological Science and Medical Engineering, Southeast University, Nanjing 210096, PR China.

***Correspondence Author**

Qianqian Jiang, School of Food and Biological Engineering, Yantai Institute of Technology, Yantai, China. E-mail: [jiangqianqian@yitsd.edu.cn](mailto:jiangqianqian@yitsd.edu.cn;)

Xuejun Kang, School of Biological Science and Medical Engineering, Southeast University, Nanjing 21009 6, PR China. E-mail: [xjkang64@163.com](mailto:xjkang64@163.com)

**1 │ Analyses of Characterization Instruments**

Nanofibers were characterized using Hitachi Regulus 8100 Scanning Electron Microscope (Hitachi, Japan), FEI Tecnai F20 transmission electron microscope (USA Thermo Company) and VERTEX 80v Fourier Transform Infrared Spectrometer (Bruce GMBH, Germany).

**Instrumental Nnalyses:**

**SEM:** By using electron beam and electron lens instead of light beam and optical lens, the fine structures of nanofibers are imitated at very high magnification.

**TEM:** The accelerated and concentrated electron beam is projected onto a very thin sample, resulting in solid Angle scattering, resulting in a different light and dark image, which will be enlarged and focused on the imaging device to display the internal structure and ultrastructural details of the nanofibers.

**FTIR:** After the interference light containing sample information passes through the detector, the signal is processed by Fourier transform, which can be used to identify the characteristic absorption peaks of different chemical groups in the nanofibers.

**2 │ Optimization of Spinning Parameters**

**TABLE S1** │ Optimization of spinning parameters.

| Item | Argument | Experimental result |
| --- | --- | --- |
| electrostatic high voltage voltage  (kV) | 17 | There are spinning droplets, the needle is easily blocked |
|  | 19 | The spinning solution is uniformly sprayed without clogging, and the dense nanofibers can be spun stably |
|  | 21 | The spun fibers are uniform in shape, but have poor mechanical strength |
|  | 23 | The spun fibers break very easily |
| Receiving distance (cm) | 10 | The spun nanofibers are relatively clustered and have poor dispersion |
|  | 15 | The nanofibers are centered in the middle of the receiver and gradually dispersed around it |
|  | 20 | The nanofibers are dispersed throughout the receiver, but some fibers are difficult to collect due to the distance between the needle and the receiver |
| Spinning fluid advance speed (mL/h) | 1.0 | Needles tend to get clogged |
|  | 1.5 | The surface shape of nanofibers is uniform, and the tip of the needle is not blocked |
|  | 2.0 | The spinning solution drops and some spinning solution is difficult to spin into fiber |

The single factor variable method was used to optimize the parameters of electrostatic spinning, including the number of electrostatic high voltage, the receiving distance and the speed of spinning fluid to determine the best spinning parameters. The design and results of the optimization experiment are shown in Table S1. According to the experimental results, the following spinning parameters are determined as the subsequent spinning conditions: the electrostatic high voltage voltage is 19 kV; The distance from the spinning needle to the receiving screen is 15 cm; The advancing speed of the spinning fluid is 1.5 mL/h.

Note: The experimental material was 10%PS

**3. │ Evaluation of the Green Characteristics and Practicability of the Method**

**TABLE S2** │ Analytical greenness index evaluation table for sample preparation

| # | Criterion |  | Score | Weight |
| --- | --- | --- | --- | --- |
| 1. | Sample preparation placement | On-line/In situ | 0.66 | 1 |
| 2. | Hazardous materials | 0.912 [g or mL] | 0.35 | 5 |
| 3. | Sustainability, renewability, and reusability of materials | Materials are not sustainable or renewable, but are used SEVERAL TIMES | 0.50 | 2 |
| 4. | Waste | 1.712 [g or mL] | 0.54 | 4 |
| 5. | Size economy of the sample | Mass or volume of the sample:0.5 [g or mL] | 0.77 | 2 |
| 6. | Sample throughput | 48 [samples/h] | 0.91 | 3 |
| 7. | Integration and automation | Sample prep. steps: 3 steps, Semi-automated systems | 0.38 | 2 |
| 8. | Energy consumption | 8.93 [W] | 1.00 | 4 |
| 9. | Post-sample preparation  configuration for analysis: | Spectrophotometry, surface analysis techniques, voltammetry, potentiometry, etc. | 0.75 | 2 |
| 10. | Operator's safety: | 3 hazard | 0.25 | 3 |

**TABLE S3** │ Detailed evaluation indicators of Complex Modified-GAPI

| SAMPLE PREPARATION | |
| --- | --- |
| 1- Collection: | On-line or at-line |
| 2- Preservation: | Chemical or physical |
| 3- Transport: | None |
| 4- Storage: | Under normal conditions |
| 5 - Type of method: | Extraction required |
| 6 - Scale of extraction: | Nano-extraction |
| 7 - Solvents/reagents used: | Non-green solvents/reagents used |
| 8- Additional treatment: | Simple treatments (clean up, solvent removal, etc.) |
| REAGENT AND SOLVENTS | |
| 9-Amount: | <10 mL (<10 g) |
| 10 - Health hazard: | Slightly toxic, slight irritant; NFPA health hazard score = 0 or 1 |
| 11 - Safety hazard: | Highest NFPA flammability or instability score of 0 or 1. No special hazards |
| INSTRUMENTATION | |
| 12 - Energy: | ≤0.1 kWh per sample |
| 13- 0ccupational hazard: | Hermetic sealing of analytical process |
| 14- Waste: | 1-10 mL (1-10 g) |
| 15 - Waste treatment: | Degradation, passivation |
| 16- QUANTIFICATION: | Yes |
| YIELD AND CONDITIONS | |
| I- Yield: | >89% |
| II - Temperature/time: | Room temperature, <1 h |
| RELATION TO GREEN ECONOMY | |
| III- Number of rules met: | 5-6 |
| REAGENTS AND SOLVENTS | |
| IVa - Health hazard: | Slightly toxic, slight irritant; NFPA health hazard score of 0 or 1 |
| IVb - Safety hazard: | Highest NFPA flammability, instability score of 0 or 1. No special hazards |
| INSTRUMENTATION | |
| Va - Technical setup: | Common setup |
| Vb- Energy: | ≤0.1 kWh per sample |
| Vc - Occupational hazard: | Hermetization of the analytical process |
| WORKUP AND PURIFICATION | |
| Vla - Workup and purification of the end product: | None or simple processes |
| VIb - Purity: | >98% |
| 27.E-FACTOR | - |
| This application is for research purposes. | |

**TABLE S4** │ Detailed evaluation indicators of Blue Applicability Grade Index

| 1.Type of analysis | Quantitative and confirmatory |
| --- | --- |
| 2.Multi-or single-element analysis | Single Element |
| 3.Analytical technique | Simple instrumentation available in most labs (UV, HPLC-UV, HPLC-DAD, UHPLC, FAAS, ETAAS, ICP-OES, GC-FID etc.) |
| 4.Simultaneous sample preparation | 2-12 |
| 5.Sample preparation | Miniaturized extraction sample preparation (SPME, DLLME, MEPS, SBSE, d-SPE, FPSE, etc.) |
| 6.Samples per h | >10 |
| 7.Reagents and materials | Commercially available reagents not common in QC labs (derivatization reagents, SPE cartridges, SPME fibers, etc.) |
| 8.Preconcentration | Preconcentration required. Legislation criteria met after complicated stages (e.g. extraction, evaporation, and reconstitution) |
| 9.Degree of automation | Semi-automated with common devices (e.g. HPLC autosampler) |
| 10.Amount of sample | 501-1000 μL (or mg) bioanalytical samples; 51-100 mL (or g) food/environmental |

**4 │ Direct View of Electrospinning Nanofibers**

The direct image of 10%PS electrospun nanofibers obtained by electrostatic spinning is shown in FigureS1 a and a ', and the fiber film is uniform and compact.


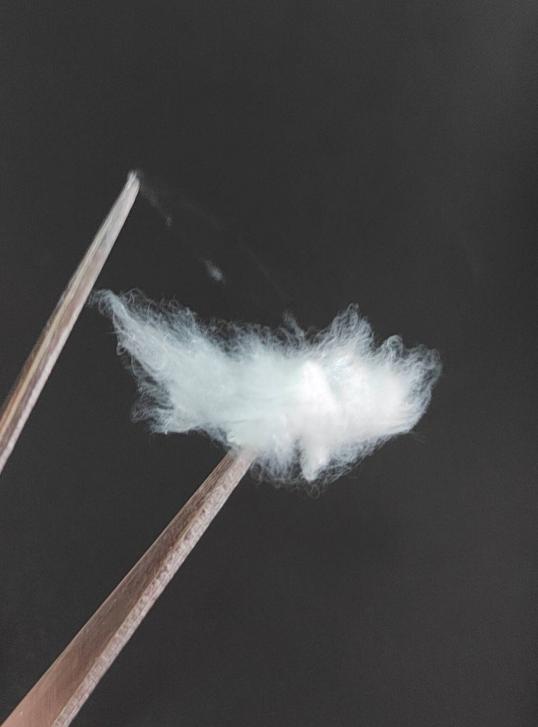


**a’**


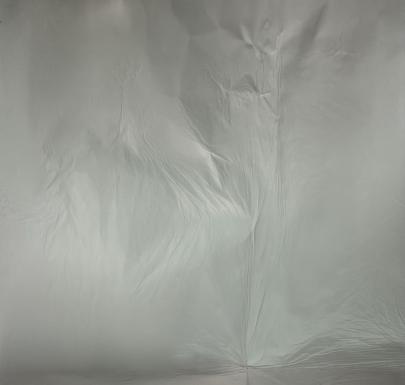


**a**

**FIGURE S1** │ (a) The image of 10%PS membrane on a receiving device; (a’) The detail image of 10% PS.

**5 │ Actual Images of SPE Processor and SPE Column**

First, nanofibers are filled to the tip of SPE empty column to prepare nanofiber solid phase extraction column. The column is then assembled into a semi-automatic array SPE processor that can preprocess 12 samples simultaneously. Figure S2 shows an SPE array processor and an empty SPE column.


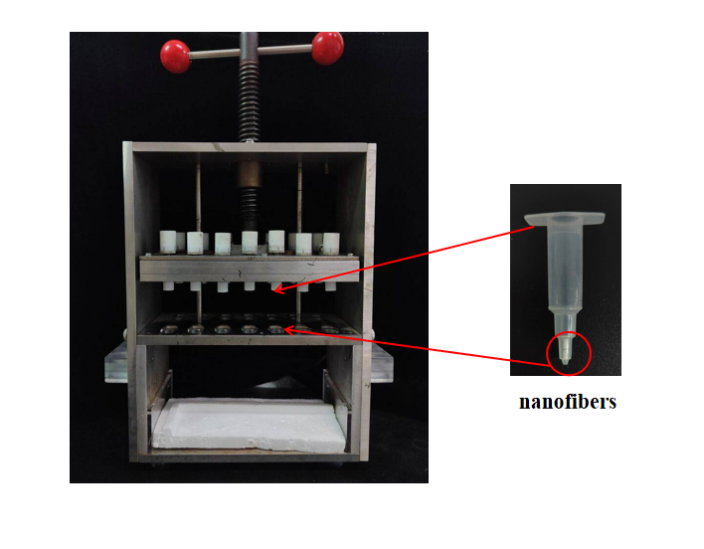


**FIGURE S2** │ Actual images of SPE processor and SPE column.

**6 │ Determination of the Detection Wavelength of isoflavones**

**FIGURE S3** │ Ultraviolet spectra of 0.05, 1, 2 µg/mL isoflavones solution.


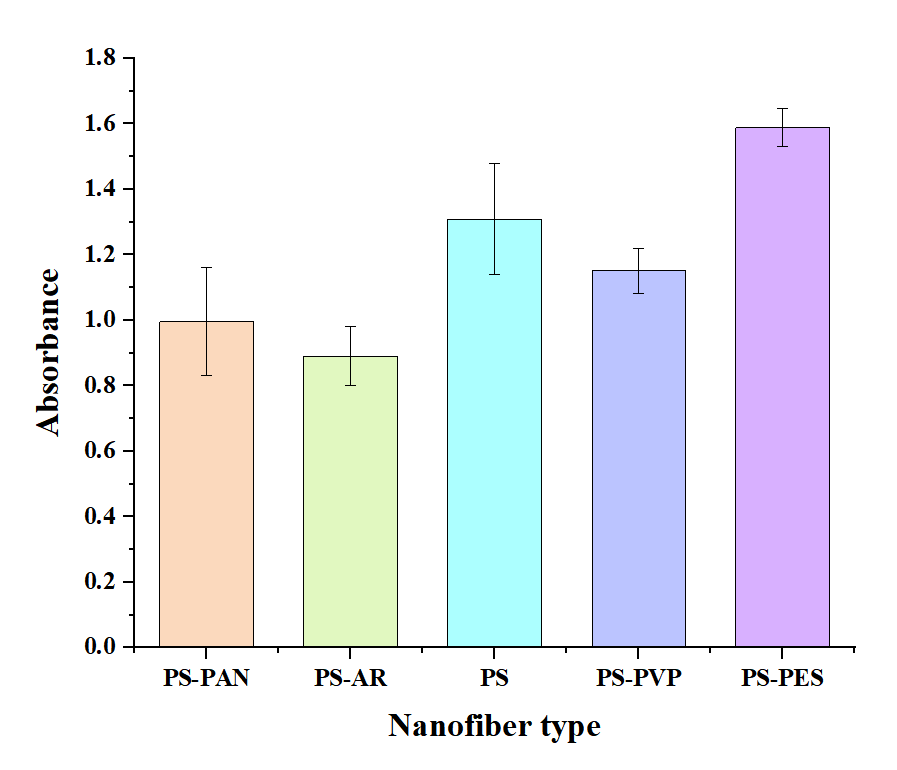
**7 │ Optimization of Solid Phase Extraction Conditions**

**FIGURE S4** │ Effect of fiber type.

**
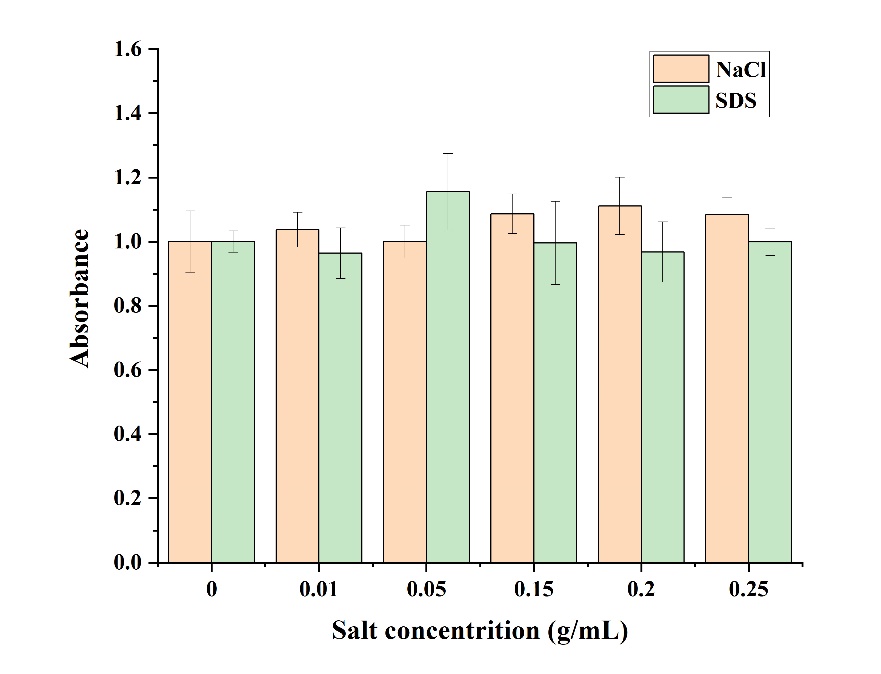
FIGURE S5** │ Influence of salt ion type and concentration.


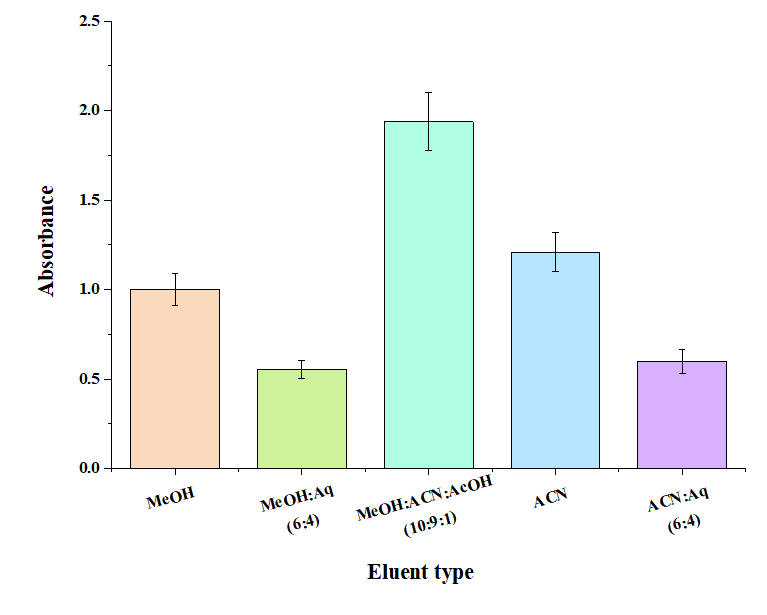


**FIGURE S6** │ Influence of eluent type.


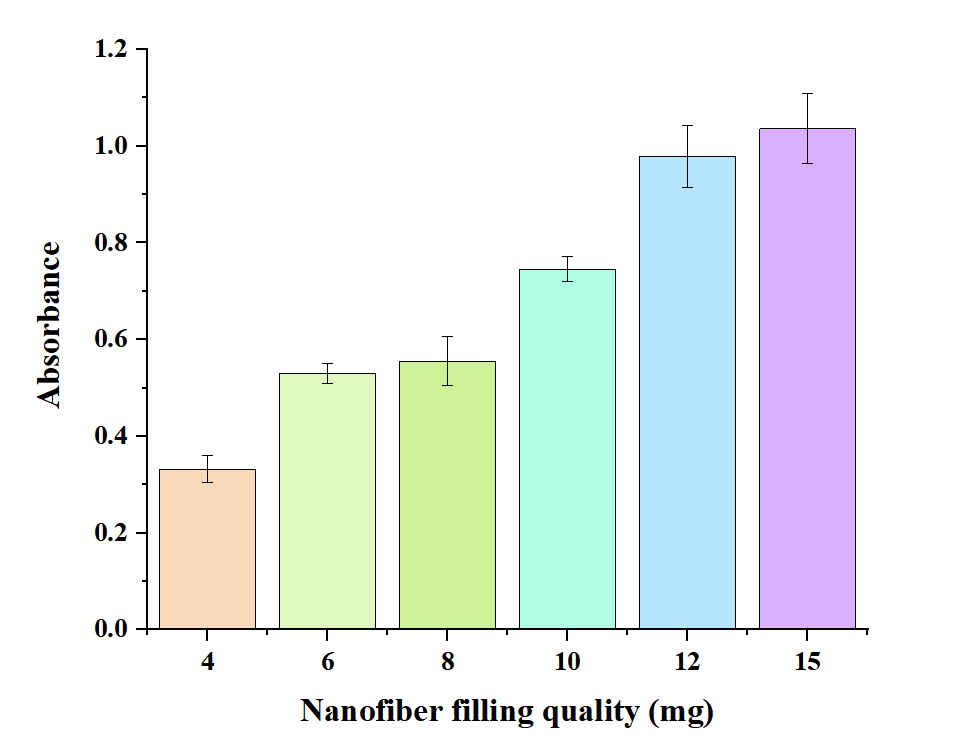


**FIGURE S7** │ Influence of fiber filling amount.


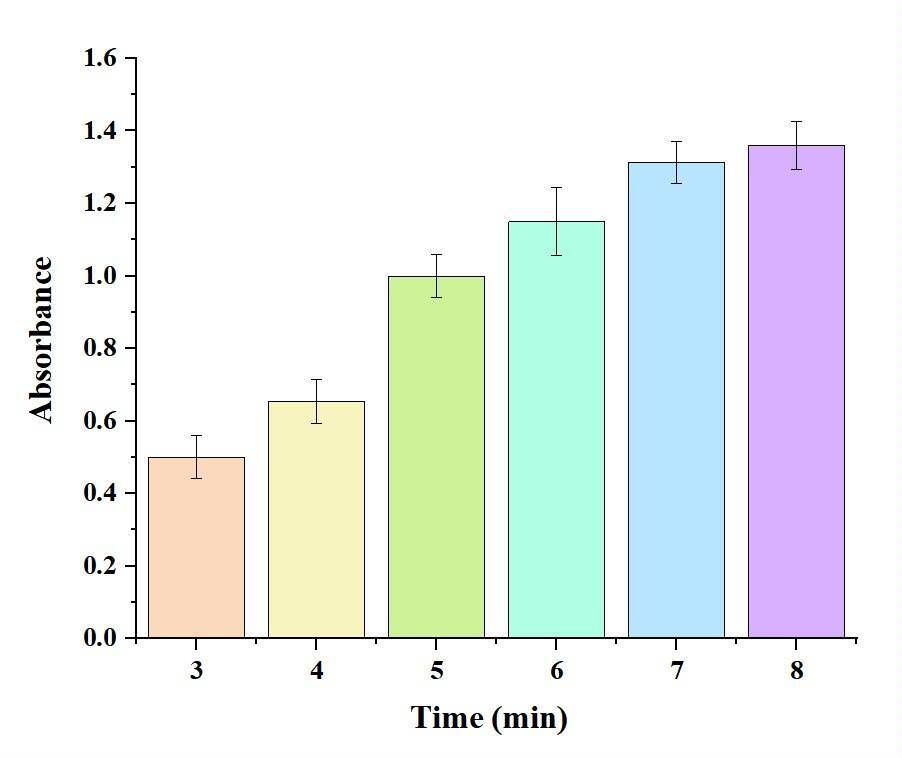


**FIGURE S8** │ Influence of extraction time.


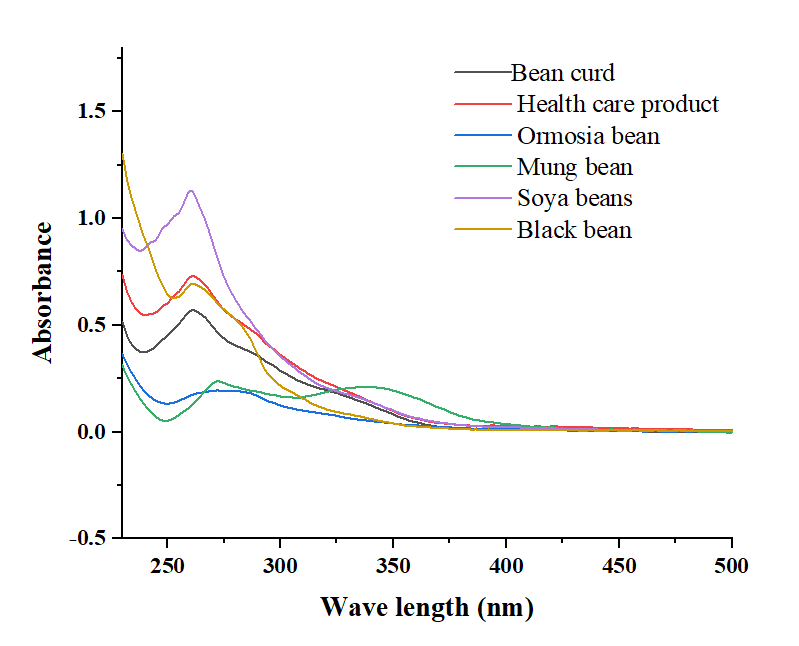
**FIGURE S9** │ Ultraviolet spectra of actual samples
